# Toward a unified view of the speed-accuracy trade-off

**DOI:** 10.3389/fnins.2015.00139

**Published:** 2015-04-28

**Authors:** Dominic Standage, Da-Hui Wang, Richard P. Heitz, Patrick Simen

**Affiliations:** ^1^Department of Biomedical and Molecular Sciences, Queen's UniversityKingston, ON, Canada; ^2^Department of Systems Science, Beijing Normal UniversityBeijing, China; ^3^Department of Psychology, Vanderbilt UniversityNashville, TN, USA; ^4^Department of Neuroscience, Oberlin CollegeOberlin, OH, USA

**Keywords:** speed-accuracy trade-off, decision making, bounded integration, decision neuroscience, neural mechanisms of cognition

Hasty decisions often lead to poor choices, whereas accurate decisions are ineffective if they take too long. Thus, good choices require cognitive mechanisms to determine the appropriate balance between speed and accuracy, and to control decision processing accordingly. This balance is referred to as the speed-accuracy trade-off (SAT) and the mechanisms by which it is determined and imposed are the subject of this Frontiers Research Topic. Given the near-ubiquity of the SAT across species and experimental tasks, it is not surprising that a wide range of methods have been used to investigate it. Our aim is to provide a unified view of the SAT in light of this diverse methodology. Computationally, decision making and the SAT are well characterized by the framework of *bounded integration*, providing a solid foundation for this view. Under this framework, noisy evidence for the available choices is added up (integrated) until the running total for one of them reaches a criterion (the bound). The SAT is readily controlled by the bound, where a higher bound favors accuracy at the expense of speed and *vice versa*. In this collection, we use bounded integration as a reference point for considering the factors that determine the optimal balance between speed and accuracy, the interpretation of behavior by different models from this general class, and the neural implementation of the computations captured by these models. Articles herein further consider conditions under which the above descriptions of the SAT and bounded integration do not explain behavior, and the utility of the SAT for manipulating the context of decisions.

The review by Heitz (2014) describes the history of the SAT as a quantifiable behavioral phenomenon and provides a critical appraisal of methodologies for its study. His historical account describes the shaping of decision theory by the SAT, a perspective that nicely sets up the original research article by Ivanoff et al. (2014), who used SAT methodology to investigate *spatial compatibility* effects, that is, how the respective locations of stimuli and responses can influence behavior. They found that SAT manipulations can systematically promote or impede the efficacy of stimulus-response mappings.

Stone (2014) investigated the relationship between speed and accuracy in his original research article, reasoning that the information gained by the observation of evidence should be reflected in both the speed and accuracy of decisions. By fitting a bounded integration model to experimental data, he used model parameters to estimate the mutual information between perceptual evidence and speed, and between perceptual evidence and accuracy. These measures provide bounds on the information gained by the observation of evidence and were used to calculate the smallest detectable change in the strength of evidence.

Salinas et al. (2014) reviewed recent studies of perceptual decisions under extreme time pressure. In this context, the respective contributions of perception and motor planning to choice behavior can be distinguished from one another, quantifying how the former guides the latter. These experiments showed that perceptual information can accelerate or decelerate the competition between ongoing motor plans, revealing the SAT as the combined effect of multiple adjustments to decision processing, not a monolithic phenomenon.

The isolation of perception from motor planning under extreme time pressure (Salinas et al., 2014) is manifest in the independence of accuracy from decision time, which constitutes a violation of the SAT. Another well-known violation is the improvement in speed *and* accuracy while learning a task. This improvement is readily captured by increasing a parameter that loosely corresponds to the difference in strength between sources of evidence, often referred to as “drift.” In effect, learning mimics a decrease in task difficulty. In their original research article, Zhang and Rowe (2014) used a bounded integration model to investigate the effects of speed and accuracy emphasis during and after learning. Under accuracy emphasis, increasing the bound and the drift captured subjects' behavior at the beginning of learning, whereas only an increase in the bound captured behavior after learning. Their results suggest that learning and speed-accuracy emphasis differentially influence decision processing on different timescales.

It is widely accepted that the objective of the SAT is to optimize decisions in terms of reward rate, that is, decision makers aim to maximize the pay-off of the task at hand. Three original research articles in the collection investigated optimal decision making, each considering a different set of conditions and corresponding strategies. Khodadadi et al. (2014) considered the case of a limited time interval, during which decision makers can make as many (or as few) decisions as they wish. This task can be formulated as a search for the reward-maximizing bound in a given condition. Khodadadi et al. (2014) took a *reinforcement learning* approach to this problem, specifying a set of conditions, each corresponding to a configuration of task constraints, e.g., the difficulty of the task, the magnitude of reward and so forth. In the terminology of reinforcement learning, each condition is a “state” and the bound that maximizes the reward rate in that condition is its “action” under the optimal “policy.” Their model took a conservative strategy, choosing a high, sub-optimal bound in the early stages of learning, before lowering it with experience to achieve optimality. This result is a testable prediction for behavioral experiments.

Karsilar et al. (2014) investigated decisions with deadlines, in which the optimal strategy is to reduce the bound during each decision. This strategy ensures that decisions are always made by the deadline, at a cost of lower accuracy. As such, decision makers have to estimate the upcoming deadline and have to account for the variability in these estimates. Crucially, models that implement this strategy predict that accuracy will decline to near-chance levels as the deadline approaches. Karsilar et al. (2014) tested this prediction with a perceptual choice task, finding that subjects' performance did not decline to chance levels near the deadline, and that a slight decline did not relate to timing variability. Furthermore, subjects' behavior was captured by a standard bounded integration model. These results suggest that perceptual decisions are too short for within-trial adaptation of the neural mechanisms captured by the bound.

As described above, the fundamental principle of bounded integration is that the effect of *within-trial* noise can be limited by integrating evidence. Goldfarb et al. (2014) compared several bounded integration models with a popular model that does not include within-trial noise, in which decision-time variability and error rates are determined only by *between-trial* noise, i.e., parameter values that vary from trial to trial. Their study focused on reward-maximization tasks, in which task difficulty is held constant for a block of trials and subjects try to earn as much reward as possible, i.e., they try to optimize the trade-off between speed and accuracy for a given task difficulty. Significant differences were found between the classes of model, especially on difficult tasks. As such, the models provide different interpretations of behavior as task difficulty increases.

The issue of optimality is further considered by Pirrone et al. (2014), who took an evolutionary perspective in their opinion article. They argued that in most real-world decisions, each of the alternatives offers some quantity of reward (e.g., deciding between food items), whereas the dominant experimental approach to date has been to reward a single alternative only. Therefore, they suggest that most natural decisions are value-based, necessitating a *speed-value* trade-off, optimized by natural selection. They formalized this optimization problem and argued that bounded integration models that optimize the SAT can only account for value-based decisions if their parameter values are assigned on a case-by-case basis, limiting their generality.

The hypothesis and theory article by Standage et al. (2014b) questioned the commonly held view that the bound is implemented by the rate of decision-correlated neural activity at the time of commitment to a choice, as well as the view that the difference between this rate and a “baseline” rate controls the SAT. Using a model derived from biophysical considerations, they showed that these views may be inconsistent with widely-held principles of cortical computation, which account for the SAT. According to their hypothesis, the behavior of the bound is well-approximated by an emergent property of cortical dynamics, but not by the aforementioned difference in firing rates.

The SAT has long been investigated as a behavioral phenomenon, but studies addressing its neural basis are a recent development. Standage et al. (2014a) reviewed hypotheses on the neural basis of the SAT, considering three general mechanistic categories: modulation of the encoding of evidence under speed and accuracy emphasis, modulation of the integration of encoded evidence, and modulation of the amount of integrated evidence sufficient to make a choice. Thus, their review is structured by the principles of bounded integration, but they focused on models addressing the neural implementation of these principles, and on the explanations offered by these models for a growing body of neuroimaging and electrophysiological data. This convergence of neural and behavioral data with models at different levels of abstraction is exemplary of interdisciplinary neuroscience, and suggests a productive future for the mechanistic study of decision making, the SAT and cognition.

We believe this collection of articles provides a useful reference for future SAT research, with review articles to direct readers to relevant work in the literature, opinions and hypotheses on the interpretation of topical methodologies and data, and original research articles that make important advances in the field. Moreover, we believe that bounded integration has successfully provided a unifying framework for the collection, supporting the systematic consideration of the SAT under different methodologies, at different levels of abstraction, and from different perspectives. A complete theory of decision making must explain the SAT. We hope this Research Topic makes a valued contribution toward this fundamental goal of cognitive neuroscience.

## Conflict of interest statement

The authors declare that the research was conducted in the absence of any commercial or financial relationships that could be construed as a potential conflict of interest.
